# Assessing colonoscopic inspection skill using a virtual withdrawal simulation: a preliminary validation of performance metrics

**DOI:** 10.1186/s12909-017-0948-6

**Published:** 2017-07-12

**Authors:** Christine M. Zupanc, Guy M. Wallis, Andrew Hill, Robin Burgess-Limerick, Stephan Riek, Annaliese M. Plooy, Mark S. Horswill, Marcus O. Watson, Hans de Visser, David Conlan, David G. Hewett

**Affiliations:** 1 School of Human Movement and Nutrition Sciences, The University of Queensland, Brisbane, Australia; 2 School of Psychology, The University of Queensland, Brisbane, Australia; 3 Clinical Skills Development Service, Metro North Hospital and Health Service, Brisbane, Australia; 4 Minerals Industry Safety and Health Centre, The University of Queensland, Brisbane, Australia; 5 School of Medicine, The University of Queensland, Brisbane, Australia; 6 Australian e-Health Research Centre, CSIRO, Brisbane, Australia

**Keywords:** Virtual reality, Training, Skill assessment

## Abstract

**Background:**

The effectiveness of colonoscopy for diagnosing and preventing colon cancer is largely dependent on the ability of endoscopists to fully inspect the colonic mucosa, which they achieve primarily through skilled manipulation of the colonoscope during withdrawal. Performance assessment during live procedures is problematic. However, a virtual withdrawal simulation can help identify and parameterise actions linked to successful inspection, and offer standardised assessments for trainees.

**Methods:**

Eleven experienced endoscopists and 18 endoscopy novices (medical students) completed a mucosal inspection task during three simulated colonoscopic withdrawals. The two groups were compared on 10 performance metrics to preliminarily assess the validity of these measures to describe inspection quality. Four metrics were related to aspects of polyp detection: percentage of polyp markers found; number of polyp markers found per minute; percentage of the mucosal surface illuminated by the colonoscope (≥0.5 s); and percentage of polyp markers illuminated (≥2.5 s) but not identified. A further six metrics described the movement of the colonoscope: withdrawal time; linear distance travelled by the colonoscope tip; total distance travelled by the colonoscope tip; and distance travelled by the colonoscope tip due to movement of the up/down angulation control, movement of the left/right angulation control, and axial shaft rotation.

**Results:**

Statistically significant experienced-novice differences were found for 8 of the 10 performance metrics (*p*’s < .005). Compared with novices, experienced endoscopists inspected more of the mucosa and detected more polyp markers, at a faster rate. Despite completing the withdrawals more quickly than the novices, the experienced endoscopists also moved the colonoscope more in terms of linear distance travelled and overall tip movement, with greater use of both the up/down angulation control and axial shaft rotation. However, the groups did not differ in the number of polyp markers visible on the monitor but not identified, or movement of the left/right angulation control. All metrics that yielded significant group differences had adequate to excellent internal consistency reliability (α = .79 to .90).

**Conclusions:**

These systematic differences confirm the potential of the simulated withdrawal task for evaluating inspection skills and strategies. It may be useful for training, and assessment of trainee competence.

## Background

The diagnosis and prevention of colorectal cancer via colonoscopy relies on the quality of mucosal inspection, which is primarily undertaken during the withdrawal phase of the procedure. The endoscopist’s task is to manipulate the colonoscope tip while withdrawing the instrument from the colon, systematically inspecting the colonic mucosa to identify cancers and potential cancer precursors, including adenomatous polyps. Depending on the size of the polyps, average adenoma miss rates ranging from 2% (≥10 mm polyps) to 26% (1-5 mm polyps) have been reported in tandem studies [1]. Rates of post-colonoscopy colorectal cancer are strongly correlated with endoscopists’ adenoma detection rates and it has been suggested that, in many instances, the cancers or their precursors were reached by the endoscopist but not visualized adequately [2–4]. Polyp detection rates are known to vary substantially between endoscopists and to improve with training [5–7].

Attempts to explain variability in detection rates have focused on the time taken to perform the withdrawal phase of the procedure under the assumption that shorter withdrawal times yield poorer detection rates. However, early research supporting the imposition of a minimum withdrawal duration [8] has been countered by a failure to replicate its positive impact [9]. A focus on withdrawal time alone is likely to be insufficient, and other aspects of the endoscopist’s technique are likely to be relevant [10–12]. For example, significant improvements in adenoma detection rates have been reported after implementing minimum withdrawal times in conjunction with a range of other changes to inspection techniques (i.e. ensuring adequate insufflation, examining flexures and proximal sides of haustral folds, suctioning residual liquid, repetitive examination of colonic segments, and torque maneuvers to better visualize regions between haustral folds) [13].

Because of the many factors that may affect performance of the inspection task, it is not obvious how performance can be adequately assessed during live colonoscopy. One alternative is the use of virtual simulation. Simulators offer the possibility of objectively and automatically quantifying many of the factors relevant to effective inspection, and allowing trainees to be assessed on standardized cases. A variety of virtual reality colonoscopy training simulators are available which report a range of quantitative data describing inspection performance, such as the percentage of the mucosa visualized, withdrawal time, time in “red-out”, and the polyp detection rate [14–16]. However, the utility of such measures remains largely untested.

This study uses a virtual colonoscopy simulator with a highly realistic mucosal surface appearance and the unique facility to simulate the withdrawal phase of colonoscopy in isolation, to compare experienced endoscopists and novices on a wide range of performance metrics to preliminarily assess the validity of these measures to describe inspection quality. The study has broad implications for the characterization and assessment of mucosal inspection performance for use during both training and assessment.

## Methods

Experienced endoscopists and novices completed a colonoscopic inspection task during four simulated cases (one practice case and three test cases) in which they searched the mucosa for “polyp markers” while withdrawing the colonoscope, and the simulator generated a range of metrics to describe their performance. Comparing the groups allowed us to evaluate whether the measures that the simulator reports correspond to the users’ levels of expertise in live colonoscopy (given that we would expect the experienced colonoscopists to perform better than the novices if the metrics do in fact measure aspects of skilled colonoscopic inspection performance). This particular technique is often used to establish preliminary evidence that the performance measures generated by a simulation device have “construct validity”; that is, that they measure what they purport to measure [17–20].

### Participants

A power analysis was conducted using G*Power 3.1.2 [21] to determine the minimum sample size required for the study (based on a *t*-test for the difference between two independent group means). We expected large experienced-novice differences in which the experienced endoscopists would out-perform the novices by at least one standard deviation. G*Power indicated that a minimum total sample of 28 participants was required to detect an effect size of *d* = 1 with 80% power and alpha set at .05 (one-tailed). We therefore aimed to recruit at least 14 participants to each group (i.e. experienced colonoscopists and endoscopy novices), plus an additional four participants per group to allow for potential exclusions. Ultimately, there was only one exclusion (i.e. an experienced endoscopist who withdrew from the study part-way through the test session), but we were unable to recruit 14 experienced endoscopists during the four-month study period (November 2010 to March 2011). Nevertheless, an additional power analysis revealed that, even with an allocation ratio of 0.6:1, a total sample of 28 participants (i.e. 10 experienced endoscopists and 18 novices) was still sufficient to detect the same effect size with 80% power.

A final sample of eleven experienced endoscopists certified with the Australian Conjoint Committee for Recognition of Training in Gastrointestinal Endoscopy (9 male, 2 female; 10 gastroenterologists and 1 colorectal surgeon; average age 48 years, range 36 to 68, *SD* = 11.3) participated in the study. On average, the endoscopists had completed approximately 12,700 colonoscopies (range 1000 to 40,500, *SD* = 15,400) and had 14 years of colonoscopy experience without supervision (range, 3 to 35, *SD* = 12.08). Eighteen medical students (11 female, 7 male; average age 26 years, range 21 to 35, *SD* = 4.2) also participated. All were either first or second year medical students at The University of Queensland, and had no prior experience with colonoscopy.

### Simulation

The Australian Commonwealth Scientific and Industrial Research Organisation (CSIRO) Colonoscopy Simulator [22] was used for the study. The CSIRO Colonoscopy Simulator is of particular interest because: (i) it permits the withdrawal phase to be carried out in isolation (i.e. an insertion phase does not need to be completed first) which avoids experience-level comparisons of inspection performance being confounded by insertion performance differences; (ii) the colon models have a highly realistic mucosal surface appearance; (iii) cases can be configured by the researcher to provide differing levels of difficulty, reducing the likelihood of ‘ceiling effects’ for experienced endoscopists; and (iv) the simulator reports a variety of colonoscope handling metrics, such as total axial rotation and thumb-wheel movement measures.

**Fig. 1:**
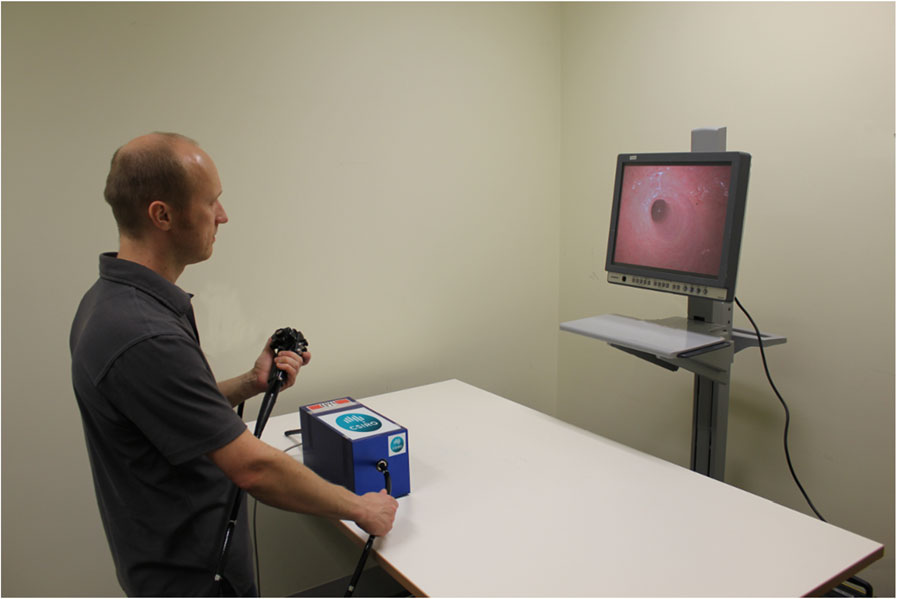
The CSIRO Colonoscopy Simulator

The CSIRO Colonoscopy Simulator (Fig. 1) incorporates a computer-generated virtual environment with a highly realistic luminal surface displayed on a computer monitor screen with a refresh rate of 30 Hz, providing a view similar to that seen via a standard endoscopy system during real colonoscopy. In the present study, the software was run on an Asus G60 J notebook computer running Windows 7 with an onboard NVIDIA GeForce GTX 260 M graphics card. The controller is a modified clinical colonoscope that includes optical encoders for monitoring the rotational motion of the two tip-control knobs [22]. During simulation, the colonoscope is inserted into a haptic device developed at the Ecole Polytechnique Fédérale de Lausanne [23]. This device, which is connected to the computer via a dedicated USB 2.0 link, monitors the colonoscope’s linear position and angle of axial rotation with an accuracy of 0.2 mm and 0.18 degrees at a rate of 100 Hz. In the study, the monitor screen was located behind the haptic device, such that the central vertical axis of the screen was approximately 30 cm to the right of the “anus” of the device.

**Fig. 2:**
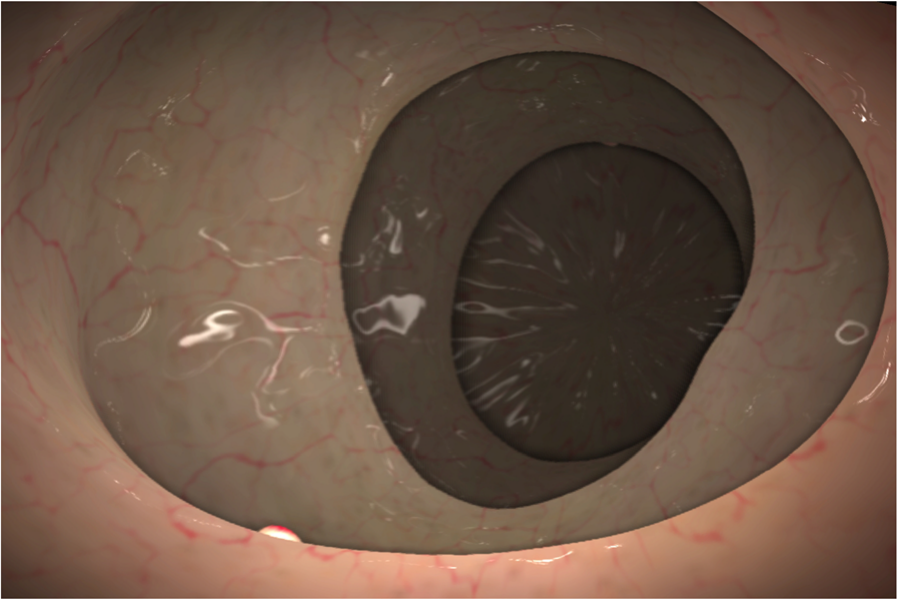
Simulated colonic mucosa, folds, and a small “polyp marker”

The CSIRO Colonoscopy Simulator allows specific cases to be created via a comprehensive set of colon model editing tools. Four colon models were created including a practice colon used to familiarize participants with the task. The colons varied in gross anatomy and in the placement of the “polyp markers” that served as search targets in the study. The focus of the study was on searching behavior during withdrawal rather than polyp recognition or diagnosis. Consequently, deliberately stylized polyp markers were used to ensure that novice performance was not confounded by their relative lack of knowledge about the subtle distinguishing features of real polyps. Figure 2 is an example image showing simulated colonic mucosa, haustral folds and a small polyp marker. The colon cases specifically configured for this study are described in Table 1. The three test colons were configured to include polyp markers with a range of sizes and alternative placements, in order to provide a varying difficulty of detection within each case – making them suitable for testing search performance in both novice and experienced participants.

**Table 1:** Characteristics of the four colon case configurations used in the study

	Colon length	Number of haustral folds	Number of chambers	Number of polyp markers	Diameter of polyp markers
Case 1 (Practice)	0.85 m	51	52	24	*M* = 1.2 mm *SD* = 0.4 mm
Case 2	0.85 m	51	52	24	*M* = 1.2 mm *SD* = 0.4 mm
Case 3	0.83 m	55	57	34	*M* = 1.5 mm *SD* = 0.6 mm
Case 4	0.74 m	62	63	34	*M* = 1.3 mm *SD* = 0.3 mm

In the study, force and torque feedback were turned off and the colon was immobilized in that colonoscope interaction with the colon could only lead to local surface deformations and not deformation of the colon as a whole. The degree of tip flexion allowed by the instrumented colonoscope was somewhat constrained and participants were not able to retroflex the colonoscope. In addition, participants were informed that the colon was suitably insufflated and clean, and were instructed not to operate the air, water or suction valves.

### Procedure

All members of the novice group participated in a 30 min familiarization session held 1 to 5 days prior to their test session. During the familiarization session, the novices were first shown how to hold the colonoscope and provided with instructions on how to steer it. This component of the training took the form of two short videos (1.16 min and 1.24 min) in which techniques for tip steering and torque steering were shown and explained. The novices then practiced steering the colonoscope tip for 15 min using the CSIRO simulator’s “virtual bowl” module, which is a virtual reality replication of a validated device for assessing and training colonoscopic tip control skill [24]. In the familiarization session, as in the study itself, participants were required to move the angulation wheels with their left hand and keep their right hand on the colonoscope shaft.

All participants were tested individually in a quiet room at the university, in a hospital simulation center, or in the participant’s consulting rooms. The protocols for the test sessions were comparable for members of both participant groups. During testing, the height of the display monitor was adjusted to the operator’s eye level and the colonoscopy simulator was mounted on an examination bed or sat on a raised platform placed on the consultant’s desk.

After receiving general task instructions, each participant was required to complete the withdrawal and inspection phase for each of the four colon cases – the practice case, followed by the three test cases in order from 2 to 4. The four cases were deliberately graded in difficulty from easiest to hardest to optimize the performance of the novice group, thus ensuring that any apparent experienced-novice differences were not over-estimates. Using a consistent order also meant that every novice received the same treatment as every expert, such that we could compare performance fairly without arbitrary order effects adding noise to the data.

In all four colon cases, the participant’s task was to withdraw the colonoscope, searching the colon for varying sized polyp markers located anywhere on the simulated colonic mucosa. Each time the participant identified a polyp marker, they pressed on a foot pedal and the polyp marker disappeared to confirm that the polyp had been “tagged”. If the participant did not finish inspecting the colon within 15 min, the trial was ended. (During pilot work, it became apparent that some novices could take over an hour to complete each case. Therefore, the time limit was imposed to reduce the likelihood that fatigue might confound the results by ensuring that the entire task did not last longer than an hour.)

The purpose of the practice case was to familiarize participants with the simulation, the response mode, and the different sizes and potential locations of the polyp markers. During the practice case, examples of polyp markers representing the full range of sizes were pointed out to the participant by the researcher. Afterwards, participants were provided with brief feedback on the time that they had taken and the percentage of polyp markers found.

### Measurements

Data were recorded from the simulator at 15 Hz. The following measures were derived from the output from each test case (i.e. Cases 2 to 4), and averaged across the three test cases for each participant prior to analysis:

1. Percentage of polyp markers found;
2. Number of polyp markers found per minute;
3. Percentage of the mucosal surface illuminated by the colonoscope for 0.5 s or more;
4. Polyp markers illuminated for 2.5 s or more, but not identified by the participant (as a percentage of all polyp markers);
5. Withdrawal time;
6. Linear distance travelled by the colonoscope (i.e., the distance travelled by the colonoscope along its axis, which is equivalent to the total distance travelled by the colonoscope tip that is not attributable to movement of the angulation controls or rotation of the colonoscope shaft);
7. Total distance travelled by the colonoscope tip;
8. Distance travelled by the colonoscope tip due to rotational movement of the up/down angulation control;
9. Distance travelled by the colonoscope tip due to rotational movement of the left/right angulation control; and
10. Distance travelled by the colonoscope tip due to axial rotation (i.e. rotation of the colonoscope shaft).

### Statistical analyses

Cronbach’s coefficient α was used to assess the internal consistency of each of the 10 performance measures (which were all composites formed by averaging over the three test cases, as described above). Cronbach’s α provides an estimate of scale reliability based on the intercorrelations between response data for component items [25, 26]. In this case, the component items for each performance measure were the relevant scores (e.g. the percentage of polyp markers found) from the three test cases (i.e. Cases 2, 3, and 4). Values of α equal to or greater than 0.7, 0.8, and 0.9 may be regarded as indicating acceptable, very good, and excellent internal consistency, respectively [17, 18].

For performance measures that yielded normally distributed data, independent samples *t*-tests were calculated to compare the groups. (However, additional analyses conducted in response to a reviewer comment indicated that substituting nonparametric Mann-Whitney tests yielded an identical pattern of significant and non-significant results across measures, with all significant *p*-values below .005.) For the remaining performance measures (i.e. those where the *z-*score for skewness and/or kurtosis exceeded ±1.96), nonparametric Mann-Whitney tests were used. For each comparison, an unbiased Cohen’s *d* (*d*_unb_) was calculated as the effect size measure, based on pooled standard deviations, with 95% confidence limits added [27]. Alpha reliabilities and inferential statistics were calculated using IBM SPSS Statistics 22 (IBM Corporation, Armonk, NY, USA) with alpha set at .05., and *d*_unb_ was calculated using ESCI [28].

## Results

### Reliability

Table 2 presents the alpha reliability for each performance measure. With only one exception, the reliabilities ranged from acceptable (α = .79) to excellent (α = .94). However, reliability was poor for the percentage of polyp markers illuminated for 2.5 s or more but not identified (α = .57).

**Table 2:** Cronbach’s alpha reliability coefficients for the 10 performance measures

Performance measure	α
1. Percentage of polyp markers found	.80
2. Number of polyp markers found per minute	.84
3. Percentage of mucosal surface illuminated for 0.5 s or more	.90
4. Percentage of polyp markers illuminated for 2.5 s or more but not identified	.57
5. Withdrawal time (min)	.79
6. Linear distance travelled by colonoscope (m)	.94
7. Total tip distance travelled (m)	.87
8. Tip distance travelled due to up/down control movement (m)	.90
9. Tip distance travelled due to left/right control movement (m)	.91
10. Tip distance travelled due to axial rotation (m)	.89

### Identification of polyp markers

**Fig. 3:**
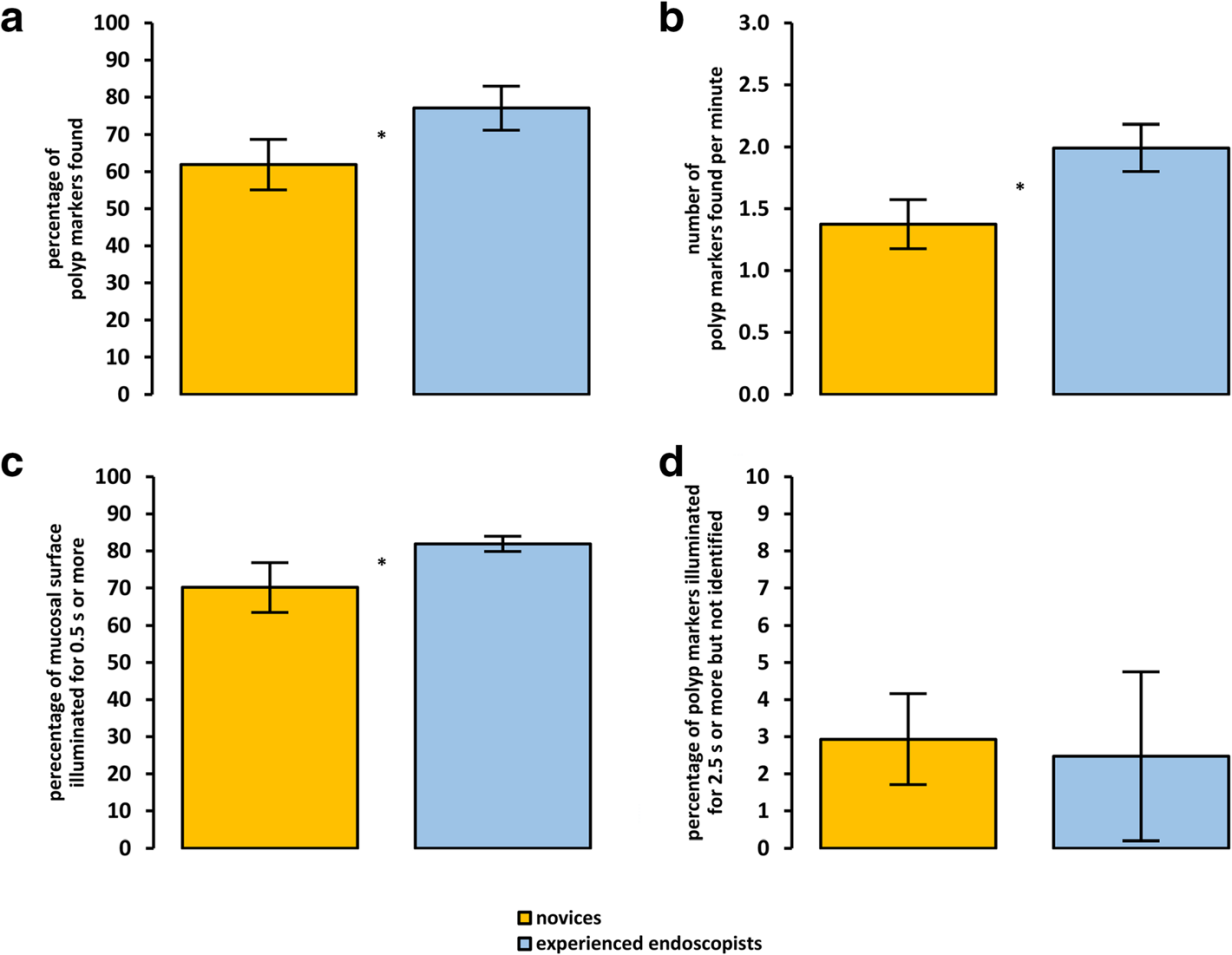
Mean (±95% CIs) performance for each experience group on measures associated with the detection of polyp markers. Specific measures are: percentage of polyp markers found (**a**); number of polyp markers found per minute (**b**); percentage of the mucosal surface illuminated (for 0.5 s or more) by the colonoscope (**c**); and polyp markers illuminated (for 2.5 s or more) but not identified by the participant, as a percentage of all polyp markers (**d**). Asterisks indicate statistically significant differences between the groups

Figure 3 and Table 3 summarize the four performance measures associated with the detection of polyp markers. Compared with the novices, the experienced endoscopists found significantly more polyp markers, *U* = 34, *z* = −2.92, *p* = .0026; *d*_unb_ = 1.22 (0.43 to 2.07), and at a faster rate, *t*(27) = 4.47, *p* = .0001, *d*_unb_ = 1.66 (0.82 to 2.58) [25, 26]. While the experienced endoscopists illuminated a larger proportion of the mucosa than the novices, *U* = 23.50, *z* = −3.39, *p* = .0003, *d*_unb_ = 1.06 (0.27 to 1.88), there was no significant difference between experienced endoscopists and novices in the proportion of polyp markers that were missed when they were visible on the monitor for 2.5 s or more, *U* = 80, *z* = −0.87, *p* = .4120, *d*_unb_ = −0.16 (−0.91 to 0.59).

**Table 3:** Descriptive statistics for the performance of each experience group on measures associated with the detection of polyp markers

Measure	Group	*M* (95% CI)	Median	*SD*	Min	Max	Range	*IQR*
(a) Percentage of polyp markers found	Novices	61.92 (55.15–68.69)	63.03	13.62	25.98	85.22	59.24	14.30
	Experienced Endoscopists	77.06 (71.16–82.96)	80.89	8.78	58.19	86.08	27.89	13.92
(b) Number of polyp markers found per minute	Novices	1.37 (1.18–1.57)	1.29	0.40	0.52	2.03	1.50	0.62
	Experienced Endoscopists	1.99 (1.80–2.18)	1.95	0.28	1.39	2.39	1.01	0.39
(c) Percentage of mucosal surface illuminated for 0.5 s or more	Novices	70.19 (63.54–76.84)	74.37	13.38	32.07	83.27	51.20	15.98
	Experienced Endoscopists	81.89 (79.86–83.93)	83.27	3.03	75.70	84.67	8.97	5.27
(d) Percentage of polyp markers illuminated for 2.5 s or more but not identified	Novices	2.93 (1.70–4.15)	2.37	2.46	0.00	6.94	6.94	4.68
	Experienced Endoscopists	2.47 (0.20–4.75)	1.39	3.39	0.00	11.03	11.03	2.94

### Movement of the colonoscope

**Fig. 4:**
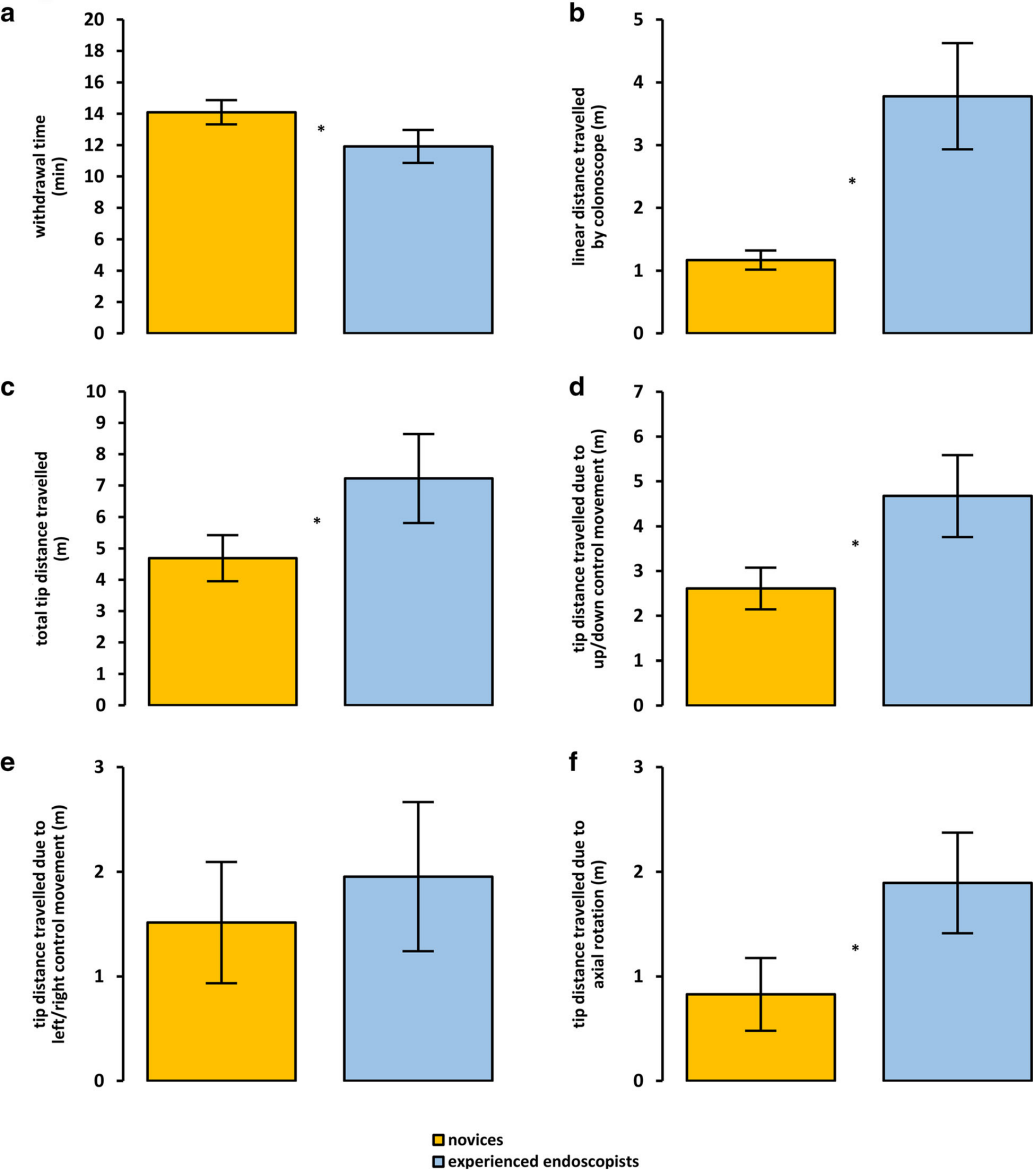
Mean (±95% CIs) performance for each experience group on measures describing the movement of the colonoscope. Specific measures are: withdrawal time (**a**); linear distance travelled by the colonoscope (**b**); total distance travelled by the colonoscope tip (**c**); and distance travelled by the colonoscope tip due to movement of the up/down angulation control (**d**), movement of the left/right angulation control (**e**), and axial rotation (**f**). Asterisks indicate statistically significant differences between the groups

Figure 4 and Table 4 summarize the measures describing movement of the colonoscope. Overall, the experienced endoscopists completed the withdrawals significantly faster than the novices, *t*(27) = 3.65, *p* = .0011, *d*_unb_ = −1.36 (−2.22 to −0.55). Nevertheless, the experienced endoscopists moved the colonoscope a significantly greater linear distance along its axis, *U* = 0, *z* = −4.45, *p* < .0001, *d*_unb_ = 3.16 (2.09 to 4.38), and also moved the tip of the colonoscope a significantly greater total distance than the novices, *t*(27) =3.82, *p* = .0007, *d*_unb_ = 1.42 (0.61 to 2.29). Compared with the novices, the experienced endoscopists moved the tip significantly further via operation of the up/down angulation control, *t*(27) = 4.86, *p* < .0001, *d*_unb_ = 1.81 (0.95 to 2.75), but not the left/right angulation control, *t*(27) = 1.02, *p* = .3179, *d*_unb_ = 0.38 (−0.37 to 1.14) The experienced endoscopists also produced significantly more tip movement due to axial (i.e. rotational) movement of the colonoscope shaft than did the novices, *t*(27) = 3.94, *p* = .0005, *d*_unb_ = 1.47 (0.65 to 2.35).

**Table 4:** Descriptive statistics for the performance of each experience group on measures describing the movement of the colonoscope

Measure	Group	*M* (95% CI)	Median	*SD*	Min	Max	Range	*IQR*
(a) Withdrawal time (min)	Novices	14.09 (13.32–14.87)	14.91	1.55	9.85	15.36	5.51	2.53
	Experienced Endoscopists	11.91 (10.86–12.97)	11.64	1.57	9.18	14.50	5.32	2.03
(b) Linear distance travelled by colonoscope (m)	Novices	1.17 (1.02–1.32)	1.19	0.31	0.62	1.55	0.93	0.53
	Experienced Endoscopists	3.78 (2.93–4.62)	3.95	1.26	2.06	5.71	3.65	2.30
(c) Total tip distance travelled (m)	Novices	4.69 (3.95–5.42)	4.88	1.48	2.63	8.39	5.75	2.02
	Experienced Endoscopists	7.22 (5.81–8.65)	7.55	2.11	4.87	10.29	5.42	4.57
(d) Tip distance travelled due to up/down control movement (m)	Novices	2.61 (2.15–3.07)	2.61	0.93	1.38	4.96	3.58	1.60
	Experienced Endoscopists	4.67 (3.76–5.58)	4.51	1.36	3.36	6.75	3.39	2.56
(e) Tip distance travelled due to left/right control movement (m)	Novices	1.51 (0.93–2.09)	1.75	1.16	0.02	4.12	4.10	1.85
	Experienced Endoscopists	1.95 (1.24–2.67)	2.14	1.06	0.22	3.61	3.38	1.64
(f) Tip distance travelled due to axial rotation (m)	Novices	0.83 (0.48–1.18)	0.61	0.70	0.12	2.34	2.23	1.02
	Experienced Endoscopists	1.89 (1.41–2.37)	1.90	0.72	0.76	2.97	2.20	1.38

## Discussion

We compared the performance of experienced endoscopists and novices completing a muscosal inspection task during a series of three simulated withdrawals using the CSIRO Colonoscopy Simulator, to provide preliminary evidence of the “construct validity” and utility of the proposed measures generated by the device. Such evidence was found for three of the four metrics that related to aspects of polyp detection, and five of the six metrics that described the movement of the colonoscope, in the form of statistically significant differences between the groups (all *p*’s < .005), coupled with large effect sizes (all *d*_unb_’s > 1). All metrics that yielded significant differences also had adequate to excellent internal consistency reliability (α = .79 to .90), further supporting the validity of these measures.

In relation to aspects of polyp detection, the experienced endoscopists found significantly more polyp markers than the novice group, and found them at a faster rate. In a real colonoscopic withdrawal, such a pattern of results might be partially explained by experienced-novice differences in polyp recognition skill [29]. However, in the present study, the task was specifically designed to test only the search component of polyp detection independent of the recognition component (which can be assessed separately [29]). Consequently, the polyp markers were deliberately stylized so that they would be relatively easy to distinguish from the mucosal surface as long as scope motion was not excessively fast and an appropriate distance from the mucosal surface was maintained. Hence, prior knowledge of the subtle distinguishing features of real polyps offered no specific advantage to the more experienced participants. That the experienced colonoscopists nevertheless found more polyp markers than the novices can be explained by the higher proportion of the mucosal surface that they illuminated. However, there was no significant difference between the groups in their ability to detect the polyp markers when they were visible on the screen, indicating that – as intended – the observed differences in detection-related metrics reflected skill disparities in colonoscope manipulation rather than visual detection.

The results for metrics describing the movement of the colonoscope highlighted group-level differences in colonoscope handling that may provide insight into some of the techniques that novices need to acquire during training. Compared with novices, experienced endoscopists completed their withdrawals more quickly, taking around 2 min less on average to complete each case. Despite this, they also moved the colonoscope a greater linear distance along its axis than the novices, indicating more use of forward movement or “pushing”. In fact, they moved the colonoscope along its axis around three times as far as the novices. The endoscopists also moved the colonoscope tip more overall (independent of shaft movement), which appears to have been achieved through greater axial rotation and more use of the up/down thumb-wheel angulation control (but not the left/right control).

It has been suggested that using particular inspection techniques, including inspection behind internal colon structures and double inspection, can result in higher detection rates [10, 11, 13]. It is difficult to quantify performance of these techniques in live colonoscopy; however, the results of the present study suggest that it may be possible to do so during simulated withdrawal. For example, the CSIRO Colonoscopy Simulator’s measure of linear movement will increase if the user inspects a region of the colon more than once or “pushes down folds” (which is a common technique used by endoscopists to inspect behind them). It is likely that the use of these techniques by experienced endoscopists in the present study explains why they engaged in significantly more linear movement than novices. In contrast, the inexperienced participants would not have been aware of these techniques, so it is assumed that their linear movement during the withdrawal task would mostly have involved pulling the colonoscope back through the colon, with only a limited amount of incidental forward movement during mucosal inspection. However, it is interesting to note that, although every experienced endoscopist produced much more linear movement than any of the novices, linear shaft movement did not necessarily predict good performance on the polyp marker detection metrics within the experienced group. For example, the worst performing endoscopist (in terms of detection measures) produced by far the highest degree of linear movement (nearly twice that of any other experienced endocopist).

### Limitations

The primary limitation of the study is that, like all such devices currently available, the CSIRO Colonoscopy Simulator does not provide an entirely authentic replication of real colonoscopy. A common criticism of the simulation from the endoscopists was that, when they tried to push the haustral folds down during inspection, the simulator tended to go into “red-out”, potentially hampering their performance. Hence, it is possible that the experienced endoscopists might have performed even better relative to the novices if the simulated haustral folds had been more pliable, and further development of the simulator will be necessary if more advanced search techniques are to be investigated and assessed. In addition, several artificial constraints were placed on participants for the purposes of the study, preventing the use of retroflexion and the air, water and suction valves. Although this made the study a more focused test of basic mucosal inspection skills, and avoided penalizing novices for their lack of more advanced skills, experienced endoscopists may have performed better still (i.e. further increasing the observed experienced-novice differences) with access to their full repertoire of search techniques, such as using suction to navigate around folds.

Arguably, another limitation of the present study is that, although we assessed performance on 10 different outcome measures, we did not adjust for multiple comparisons. However, it should be noted that, even if we had applied a highly-conservative Bonferroni correction (effectively reducing the critical *p* to .005), the pattern of significant results would not have changed. Perhaps more importantly, we have not yet demonstrated that the metrics generated by the CSIRO Colonoscopy Simulator correlate with relevant real-word measures, such as clinical polyp detection rates. Although such work was beyond the scope of this preliminary validation study, it could bolster the findings and therefore remains a potentially fruitful avenue for future research.

## Conclusions

Despite the limitations outlined above, we can nonetheless conclude that the simulated mucosal inspection task described here shows promise in providing useful information about some of the technical skill characteristics required for successful colon inspection, complementing other recent attempts to more precisely characterize the bases of skilled insertion and withdrawal [30]. One implication of this work is that research questions regarding the efficacy of different inspection strategies may now be answerable using virtual simulation. More broadly, the systematic differences that were observed between experienced endoscopists and novices confirm the potential of the simulated withdrawal task for evaluating skilled inspection. The task therefore represents a valuable new tool, potentially providing both a novel adjunct to existing preclinical training methods and a means of objectively assessing competency components in colonoscopy trainees.

## Abbreviations

CSIRO: Australian Commonwealth Scientific and Industrial Research Organisation
